# Cross-Cultural Study of the Attitudes of Russian and Chinese Consumers Toward Electric Vehicles

**DOI:** 10.3389/fpsyg.2022.820584

**Published:** 2022-02-17

**Authors:** Fei Zhao

**Affiliations:** Psychology Department, National Research University Higher School of Economics, Moscow, Russia

**Keywords:** cross-culture, consumer identity, product identity, consumer loyalty, electric scooter

## Abstract

**Aim:**

The article presents the results of a study of psychological factors of consumer loyalty concerning electric vehicles. An electric scooter was used as an example of an electric vehicle. The study involved a total of 165 people in China and 150 people in Russia. The study aimed to compare the psychological characteristics of Russian and Chinese consumers based on their attitudes toward an innovative product such as the electric scooter.

**Hypotheses:**

(1) The identity of Russian and Chinese consumers and the perceived individuality of the product (electric scooter) differ significantly; (2) The proximity of the consumer's identity and the image of the product (electric scooter) enhances the positive attitude toward the product; (3) Risk strategies of consumer behavior of Chinese and Russians differ.

**Methods:**

The questionnaire included the scales: (1) “Measuring brand personality” by J. Aaker, (2) “Measuring consumer identity” elaborated by Morozova and Antononva, (3) The risk behavior scale by Vasilenko, Tkachenko.

**Results:**

(1) The first hypothesis was confirmed: there were revealed differences in the consumer behavior of the respondents of these two groups concerning in their self-perception, as well as in the perception of electric scooters. The Russians perceive an electric scooter as energetic, young, extravagant, modern, and unusual; the Chinese are down-to-earth, family-oriented, real. (2) The hypothesis that the correlation of the consumer's identity and the individuality of the product is one of the psychological factors that determine the consumer's loyalty to a product is also confirmed. (3) The Chinese consumers' identity is more close to the product individuality and their loyalty to the product is higher. (4) Risk strategies of consumer behavior of Chinese and Russians significantly differ in terms of satisfaction with choosing of vehicle, the frequency of its use, and the importance of family approval of the choice of vehicle. The results can be used in the marketing strategy development. From the scientifical perspective, these results contribute to an understanding of the consumer loyalty mechanisms associated with consumer identity.

## Introduction

Modern post-industrial society is such a set of social relations in which individual consumption occupies a key place (Ilyin, 2008). Regardless of how it relates to this phenomenon, it must be admitted that the study of consumer psychology is necessary not only and not so much for solving applied marketing problems, but for understanding the psychological factors that determine the activities of the individuals and ultimately affect society as a whole.

The ideology of consumption pleasure stimulates uncontrolled consumption, which negatively affects various spheres of society. One of these areas is the transport sector and the problem of mass motorization. In many countries, the apparent failure of car-centered transport systems is driving the development of new urban mobility concepts. There are three business models for road transport and urban mobility (Saginova and Saginov, 2019): the US model assumes the leading role of large international car manufacturers; the Chinese model is based on government funding and regulation of transport systems, assuming, first of all, development of electrified modes of transport; the European model seeks to use different approaches to the development of urban mobility, using, in particular, the activity of opinion leaders to introduce innovative vehicles into society (Seebauer, 2015).

In Russia, as in many other developing countries, where the tendencies of mass motorization intensified only in the 1990s, the problem of new urban mobility is overshadowed by more acute social problems. As a result, in megacities like Moscow, the crisis of transport systems is aggravated. However, this does not lead to the popularization of electric vehicles, as happened in China. Within the framework of this study, electric scooters are considered as an element of the concept of new urban mobility being successfully implemented in China (Cherry and Cervero, 2007; RIA Novosti, 2021).

What are the psychological reasons for the different levels of the introduction of electric scooters in the Russian and Chinese markets? The role of external factors, such as government policy, climate, and road infrastructure conditions, which determine the transport preferences of consumers, is obvious. However, there are also internal factors that influence the formation of transport attitudes.

The most significant construct in this series, is the identity of the consumer. This study focuses on the correlation between the consumer's identity and the product's personality, which, as assumed, is one of the psychological factors that determine the consumer's loyalty to the product.

According to many researchers, in modern society, consumption has become the activity through which a person constructs his identity (Ilinykh, 2011; Hamouda and Gharbi, 2013; Blagorodova, 2018). Virtually every product is viewed by the consumer as matching or not matching their identity. Such an identity is fragmented and multiple (Blagorodova, 2018), it changes depending on many factors, including the fashion for certain goods or lifestyles, on the social desirability of certain personal qualities. Endowing brands and products with personal qualities through the mechanism of personification (Perelygina, 2002; Batra et al., 2013), a person interiorizes these qualities for the second time by consuming brands and products. This allows considering the interaction of the consumer and the brand or product as a symbolic discourse that has a significant impact on a person's self-perception, firstly, and on his consumer behavior, secondly. The study of the psychological aspects of this discourse is necessary not only to competently build marketing strategies, but also to identify the mechanisms of the formation of personality identity, psychological factors of consumer behavior.

## Problem Statement

The identity of a person is formed and manifested in a cultural and ethnic context. Despite the globalization of the world economy, which dictates certain consumption standards for consumers in different countries, ethnic characteristics have a huge impact on the perception of a product or brand, and on the decision to purchase. As S. Ilyinykh notes, the significance of consumption of a particular product is determined by the system of human value orientations (Ilinykh, 2011), and these systems are different for representatives of different nationalities and different cultures. Cross-cultural studies of psychological factors influencing consumer behavior from different countries contribute to the identification of these national and cultural characteristics and are of great practical importance.

Russia and China are countries with a socialist past, which caused a certain commonality of values in these states in a certain historical period. In the late 1990s—early 2010s, the economic systems of Russia and China underwent major changes. The sphere of consumption has changed significantly and has become uncertain due to an overabundance of information about various products, brands, sellers, suppliers, etc. But although the transformation processes in these countries had a common vector of orientation, they took place in different ways: in China, with the preservation of the foundations of the way of life and the system of values; in Russia, with their radical change, which, of course, contributed to the formation of completely different models of economic consciousness and behavior (Pomozova, 2012). The above processes determined the special research interest in how cross-cultural differences in the identity of Russians and Chinese affect their perception of a product. Are the perception features determined by these differences related to product loyalty?

## Literature Review

Russian and Chinese cultures, as noted by researchers studying these nations from the point of view of philosophy and sociology, have both similarities and differences. Thus, P. Yakupov notes that Russians and Chinese are united by their readiness for life difficulties and collectivism, but they are distinguished by their attitude to work and communication features (Yakupov, 2016).

The peculiarities of mentality determine the value sphere and inevitably affect consumer behavior. In a study by E. Noskova devoted to the peculiarities of the business culture of China, the United States and Russia and their impact on consumer behavior, the influence of cultural values on consumer behavior is proved, and cross-cultural gaps in the behavior of Russian, American and Chinese consumers are found. The researchers noted that the Chinese are characterized by scrupulousness and rationalism in economic behavior, as opposed to the irrationality and risk-taking propensity of Russians (Noskova et al., 2019). At the same time, both countries are characterized by a significant influence of elements of the institutional environment on consumer behavior.

N. Danilevskaya and Wan Ning studied the advertising discourse in Russia and China on the example of automobile slogans, considering it as a reflection of nationally significant meanings. Both zones of cultural overlap and zones of a cultural discrepancy between consumers from these countries were found. For Russian consumers, the most important priority was the reliability of the car, while for Chinese consumers, both the safety of the car and its external attractiveness are important, as well as the understanding of the car as a phenomenon of scientific thought (Danilevskaya and Wan, 2014).

Cross-cultural studies comparing consumers from Russia and China point out certain points of contact between them, but at the same time, existing differences determine significant differences in consumer behavior.

This study proposes to use the obtained data when studying the psychological factors of consumer behavior in Russians and Chinese for cross-cultural analysis, as these factors consider the individuality of the product represented in the minds of the respondents and its correlation with the image of oneself.

The product for the study of this article is an electric scooter, a two-wheeled or three-wheeled vehicle with a battery-powered electric motor. Assuming a weak degree of familiarity of the respondents with this type of transportation tool, it deliberately did not present its sample for review, since our goal at this stage of the study is to obtain the attitudes toward an electric scooter that have formed among Russian and Chinese consumers at the moment. At the next stage of the implementation of the study, we plan to conduct an offline survey of respondents, while the experimental group will be given the opportunity to test drive an electric scooter. So, we are going to find out how close acquaintance with a product affects the attitude to it.

An electric scooter, as an innovative product, the introduction of which takes place differently in Russia and China (Saginov and Zavyalov, 2020). In China, this process is regulated by the state, the use of an electric scooter is encouraged, because it allows solving the problem of the crisis of transport systems in an environmentally friendly way, which leads to the formation of appropriate social norms. Huibin Du and colleagues studied the socio-psychological factors that induce consumers to buy electric vehicles from the perspective of the theory of planned behavior. They found that it is subjective norms that relate to an individual's assessment of social pressure that encourages a certain behavior that influences consumers ' intention to purchase an electric vehicle more than other factors. That means that the consumer is more likely to purchase such a vehicle if other people recommend it. In addition, the researchers found that the acceptability of government policies has a strong positive impact on the consumer's decision to buy an electric vehicle (Du et al., 2018).

European researchers studying the psychological factors that determine the use of electric transport also notice the influence of state stimulating the corresponding decision of consumers. So, the Norwegian scientist Ozlem Simsekoglua, based on his comparison of socio-demographic and psychological profiles of drivers of conventional and electric cars, as well as owners of both types of cars, formulates recommendations for optimizing measures of state support for environmental behavior, considering the characteristics of consumer motivation (Simsekoglua, 2018). A study by the Austrian scientist Sebastian Seebauer is devoted to the psychological laws of introducing an innovative product—electric transport, namely electric bicycles and electric scooters—into the market. Based on the theory of diffusion of innovations by E. Rogers, the author studies personal motives that induce early adopters to actively recommend a product not only to their familiar people around, but also to the strangers. Noting the ineffectiveness of the current policy of subsidizing electric transport, Seebauer recommends that public and private entities interested in introducing this product actively cooperate with its early followers, whom the researcher characterizes as technophiles, with strong personal norms focused on protecting the environment (Seebauer, 2015).

In Russia, this problem does not attract the attention of the government, and the use of electric scooters, accordingly, remains at the discretion of citizens. Only by 2021 the concept for the development of production and use of electric transport in Russia was developed, but it will not be approved by the Russian government until 2030 (Sazonova, 2021). However, even this document does not imply the interaction of the state with individuals since the introduction of electric transport in Russia is at a very early stage. This study in which attitudes toward electric scooters among Russian consumers are formed spontaneously, without being mediated by government regulations or the intervention of private organizations interested in introducing this product to the Russian market, is unique.

According to PWC, Russian consumers, when choosing a vehicle, consider not innovation and prestige, but comfort and a practical component (Tomorrow of the Automotive Industry., 2018). However, changes in the transport environment of large cities, including electrification of transport, are taking place all over the world, and Russia is no exception in this regard (Leal Filho and Kotter, 2015; Saginov and Zavyalov, 2020). These changes put consumers in a situation of risk and uncertainty, which is reflected in their transport preferences and consumer behavior.

The peculiarities of consumer decision-making under conditions of uncertainty were studied (Vasilenko and Tkachenko, 2016). The researchers identified the risk strategies of consumer behavior depending on the age of the respondents. Since mainly young people took part in this study, the criteria of risk behavior proposed by the authors are used to identify the characteristics of consumer behavior of respondents in a transforming transport environment. Considering cross-cultural differences, as well as differences in the institutional environment, it can be assumed that the strategies of consumer behavior among Russians and Chinese will be different.

As psychological factors of consumer loyalty to a product, we considered the personal identity of the consumer and the individuality of a product (its image represented in the consumer's mind).

Personal identity as a concept which is actively developed in many social sciences, including psychology. It is a system of ideas about oneself, life goals and values, which gives a feeling of identity and constancy of personality (Antonova, 1996). Highlighting the content, evaluative and temporal components in the structure of identity (Marcia, 1980; Breakwell, 1986), this study focuses on the content dimension of identity, which includes all the characteristics that a person uses when describing himself (Antonova and Morozova, 2015).

Consumer identity is linked to the perceived identity of a product or brand. This study relies on the brand personality model developed by Aaker (1997) to describe the specifics of consumer perception of a brand. J. Aaker understood brand personality as a set of personality traits associated with it. In her research, she relied on the concept of the “big five features.”

Both in the studies of J. Aaker herself (Aaker et al., 2001), and in many other works based on the model proposed by her (D'Astous and Levesque, 2003; Rojas-Mendez et al., 2013; Aleksandrovsky and Fomenkov, 2017; Tsoi et al., 2017) it is proved that the level of expression of brand personality traits can be measured. The quantitative assessment makes it possible to compare the images of brands represented in the minds of consumers.

The same set of personality traits, formed through the personification mechanism, can be applied to goods (Batra et al., 2013). Under the approach of J. Aaker, the predominance of one or another component in the brand personality determines the specifics of the relationship between the consumer and the brand (Antonova and Morozova, 2015). The traits prevailing in the product individuality characterize the features of its perception by consumers from a psychological point of view. The degree of conformity of the product image to consumers' self-image can be considered as an important factor of product loyalty. The hypothesis that the consistency of images of oneself and a product is associated with consumer loyalty to the brand was tested and confirmed (Antonova and Morozova, 2015).

## Methods

This study uses the model of J. Aaker to assess the individuality of a product and the identity of consumers, which plans not only to test the hypothesis about the correlation between the images of oneself and a product, but also to reveal the psychological characteristics of the perception of an innovative technically complex product by consumers of different nationalities.

### The Goal of This Study

This study is the third part of a project that investigates psychological factors that influence consumer behavior. At this stage, our goal was to compare the psychological characteristics of Russian and Chinese consumers on the example of their attitude to such an innovative, technically complex product as an electric scooter. The focus of the attention is the identity of a consumer and its correlation with the individuality of a product that exists in the mind of a consumer.

### Research Hypotheses

(1) There are psychological characteristics of Chinese and Russian consumers that affect their attitudes to electric scooter:
- The image of the product (electric scooter) is different for Chinese and Russian consumers.
- The identity of Chinese and Russian consumers has different characteristics.
(2) The proximity of the consumer's identity and the image of the product (electric scooter) enhances the positive attitude toward the product.
(3) Risk behavior among Chinese and Russian consumers differ.

### Sample

The Chinese part of the sample. In China in June 2021, the online survey through one 3rd party survey agency (www.wjx.cn) was conducted and the respondents were cross over China mainland. The study involved 165 people living in various localities of the People's Republic of China. The following sample characteristics were taken into account: gender (65 men and 100 women), age (18–57 years, 97.58% of respondents are under 50 years of age), 77% of the respondents are students. Most of the participants in the study were young people, since the problem of using electric scooters is more relevant for them.

The Russian part of the sample. The collection of data (Google questionnaire) by online survey through Facebook, Instagram, Vkontakte, WhatsApp, Telegram in Moscow and Moscow region, Russia was conducted in March 2021. The study involved 150 people living in Moscow and the Moscow region. The following sample characteristics were taken into account: gender (74 men and 76 women), age (16–60 years old, with 95.3% of respondents being people under 50 years old), 62.7% of the respondents are students.

The representativeness of the sample was ensured by the diversity of participants. In Russia, the listed social networks have their own target audiences, and the coverage of users of various social networks provided the necessary diversity. In addition, people who came from various parts of Russia live in Moscow and the Moscow region. The sample size is determined by the methodology and is considered sufficient for this design of a psychological study.

### Design

The methodological basis of this research is the concept of brand identity proposed by N. Kapferer in 1986 (Yanenko, 2016). Based on this concept J. Aaker developed a model of brand personality, which formed the basis for the “Methodology for measuring brand personality” (Aaker, 1997).

The study used a questionnaire developed while studying personal factors of preference toward electric scooters among consumers. The questionnaire includes:

(1) A socio-demographic block of questions that allows analyzing consumer groups based on such parameters as gender, age, and social status.
(2) A block of questions for analyzing the features of brand identity and consumer identity. This section includes the following methods: (1) The method of “Measuring brand identity” by Aaker (1997). The technique is based on the personification mechanism, which provokes the perception of the product as a subject (Perelygina, 2002) and assumes the assessment of the product according to the following parameters: Successful, Reliable, Spirited, Practical, Imaginative, etc. using the Likert scale (from 1 to 5), where 5 is the maximum product rating based on these parameters, and 1 is the minimum. During processing, these parameters were combined into dimensions under the model of J. Aaker: Sincerity (honest, down-to-earth, wholesome, cheerful), Excitement (daring, spirited, imaginative, up-to-date), Competence (reliable, intelligent, successful), Sophistication (charming, upper-class), Ruggedness (outdoorsy, tough).
(3) The method “Measuring consumer identity” [the method of J. Aaker with the modification by Antonova and Morozova (2015)]. Since the methodology of J. Aaker assumes the assessment of a product according to the categories by which people usually evaluate another person, the methodology “Measuring brand personality” invites respondents to rate themselves on the same dimensions.
(4) Questionnaire for identifying indicators of risk-taking consumer behavior. The questionnaire was developed on the basis of risk models of consumer behavior proposed by Vasilenko and Tkachenko (2016).

## Results

A block of questions for analyzing the characteristics of brand personality and consumer identity (the methodology “Measuring brand personality” and the methodology “Measuring consumer identity”) were tested for reliability. Alpha-Cronbach coefficient are 0.78 and 0.86, respectively, which allows to analyze the internal consistency of the methods.

### Product Image (Electric Scooter): Comparison of Chinese and Russian Consumers

Both Chinese and Russian groups noted the Ruggedness of an electric scooter. This product is perceived as sporty, dynamic, which is logical, given the purpose of this type of transport. The second significant characteristic of the image of an electric scooter in the Russian sample was Excitement, in the Chinese sample—Sincerity. Russians perceive an electric scooter as energetic, young, extravagant, modern, unusual. The Chinese perceive it as down-to-earth, family-oriented, real. This result highlights how different the perception of an electric scooter is in China and in Russia: for the Chinese consumers this product is a practical, useful thing, and for Russians it is an unusual, extravagant accessory.

When comparing the images of the electric scooter of the Russian and Chinese samples using the Student's *t* test (Mann-Whitney *U* test for independent samples), statistically significant differences were revealed on the scales of Sincerity, Competence, Sophistication and Ruggedness. This is because Chinese consumers perceive the electric scooter much more emotionally than Russian consumers, correlating it with their personality, accepting it as part of their image. On the dimension of Excitement, the respondents of both samples rate the electric scooter approximately the same.

Hypothesis 1 has been partially confirmed: the image of an electric scooter represented in the minds of Russian and Chinese respondents is significantly different. Russians perceive the electric scooter as energetic, young, extravagant, modern, unusual. The Chinese perceive it as down-to-earth, family-oriented, real.

The self-perception of Russian and Chinese respondents also revealed both similarities and differences. The leading dimensions for characterizing self-images in both groups are Ruggedness and Sincerity. Another essential characteristic of the Russians is Competence, and of the Chinese—Excitement. The Chinese celebrate their openness, while for the Russians, success is more important. In addition, the Chinese are more categorical in assessing their self-images, while the Russians are more inclined to average assessments.

When comparing the images of an electric scooter in the Russian and Chinese samples using the Student's *t*-test (Mann-Whitney U-test for independent samples), statistically significant differences were found on the Sincerity and Ruggedness dimensions. The distribution for the variable “Evaluation of oneself on the dimension of Excitement” in the Chinese sample differs from the normal, therefore, the differences between the groups for this variable were identified using the Mann-Whitney U test. The null hypothesis was rejected: the images of Russian and Chinese consumers differ significantly in terms of the Excitement dimension.

Based on primary statistics, we can conclude that respondents from China consider themselves sincerer, more emotionally vivid, and more courageous. On the one hand, this may be associated with greater self-confidence, better self-knowledge, which is a characteristic of the Chinese respondents. On the other hand, it certainly reflects the ethnic characteristics of self-perception.

The difference in self-image and the electric scooter among Chinese consumers is significantly lower (see Table 1). The Mann-Whitney U test was used to compare samples for this indicator, since the distribution of variables differs from the normal one. Significant differences between the Russian and Chinese samples were obtained using the following dimensions of the J. Aaker: Sincerity, Excitement, Competence, Ruggedness. This result confirms the result of the initial data analysis: the estimates are given by Chinese consumers to the electric scooter on the dimensions of the J. Aaker method, significantly higher. This indicates that this type of transport has become a highly involved product for Chinese consumers. The Sophistication dimension in evaluating self-images and an electric scooter is the least significant in both samples. Probably, sophistication is associated in the minds of consumers with something elegant, charming and at the same time prestigious, fashionable. All these characteristics are not associated with the image of an electric scooter.

**Table 1:** Comparison of the Russian and Chinese samples by the criteria of the difference between self-images and the image of an electric scooter on the dimensions of the J. Aaker method.

**Differences between self-images and an electric scooter**	**U Mann-Whitney**	**Z**	**Amitotic significance (2-sided)**
Sincerity	8987.50	−4.649	0.000
Excitement	9128.50	−4.422	0.000
Competence	8277.00	−5.465	0.000
Sophistication	10752.50	−2.132	0.033
Ruggedness	8872.50	−4.739	0.000

The image of oneself and the image of a product (electric scooter) is significantly closer among Chinese consumers than among Russian ones.

We can conclude that the hypothesis 2 is confirmed: the proximity of the consumer's identity and the image of the product (electric scooter) enhances the positive attitude toward the product.

### Comparison of Risk Behavior of Chinese and Russian Consumers

The transport preferences of the Chinese and the Russians are different. While most residents of Moscow and the Moscow region (over 90%) use public transport, the sympathies of the Chinese are distributed between public transport (41.8%), private cars (34.5%) and car-sharing (20.6%). When comparing the groups using the Student's *t* test, it was found that there is a statistically significant difference between the attitudes of Russian and Chinese consumers toward public transport. This is probably due to the financial status of Russian youth (namely, most of the respondents belong to this age category). It means, that they have no money for personal car or car-sharing.

We compared the Russian and Chinese samples using the criteria of risk behavior using the risk behavior scale (Vasilenko and Tkachenko, 2016). The samples differ significantly in terms of the frequency of personal transport use (Mann-Whitney U test, see Table 2). The Chinese use it more often. They are also more satisfied with their vehicle than Russians. It confirms that Russians use public transport not because they choose it but because they have no choice due to their financial status. An extremely interesting result was found in the answers to the question: “It is important for me that my choice of a vehicle is approved by the family.” For the Chinese, this indicator is significantly higher−3.6 out of 5, while for the Russians−2.6 out of 5, the difference is statistically significant. At the same time, for Russian consumers, family approval is associated with a loyalty to an electric scooter, while for Chinese consumers it is not. It is possible that this parameter is also influenced by the financial status of Russian youth, since buying an electric scooter for most Russians is a large expenditure. However, this may also be due to the perception of the electric scooter as dangerous, with a fear of innovation. The correlation between young people's consumer behavior and family approval needs the further investigation.

**Table 2:** Comparison of Russian and Chinese samples by risk behavior criteria.

**The criteria of Risk behavior**	**U Mann-Whitney**	**Z**	**Amitotic significance (2-sided)**
I often use personal transport for commuting.	7570.00	−6.096	0.000
When buying a vehicle, I always carefully weigh the pros and cons.	11205.50	−1.553	0.120
I am generally happy with my vehicles.	10199.00	−2.843	0.004
My choice of a vehicle is based on personal experience.	11585.00	−1.014	0.310
For me, it is important that my choice of a vehicle is approved by my family.	6985.50	−6.898	0.000
My friends are the source of information about various types of transport.	10971.00	−1.804	0.071
When choosing a vehicle, I listen to the advertising.	11412.00	−1.244	0.214

The samples don't significantly differ in terms of targeting one's own opinion, friends' opinion, and advertising. Thus, hypothesis 3 has been partially confirmed. Risk strategies of consumer behavior of Chinese and Russians significantly differ in terms of satisfaction with their vehicle, the frequency of its use, and the importance of family approval of the choice of vehicle.

These results are consistent with one of the main findings of Huibin Du and colleagues, that, in addition to personal opinions about electric vehicles, for Chinese consumers, positive or negative evaluations from family members, colleagues and friends are also important. This implies that there is social influence on the purchasing behavior of electric vehicles (He et al., 2014), which is consistent with Tegersen's research as well as research on the so-called Chinese mentality.

The statistically significant difference between Chinese and Russian consumers in terms of the importance of family approval of consumer behavior can be explained by the difference in value systems. So, D. Aslanov, O. Borzenko note that an orientation toward Western values is more characteristic of Russian youth (Aslanov and Borzenko, 2019), while the priority of the family is more characteristic of the Eastern culture.

No significant differences in the loyalty to electric scooters and their practical use were found between Russian and Chinese consumers: in both samples, this indicator is at a very low level. This data indicates that the formation of consumer interest in electric scooters, is at an early stage. And despite the much higher demand for electric scooters in China compared to Russia, the transport preferences of Chinese consumers are also rigid. However, the image of an electric scooter in the minds of Russian and Chinese consumers is different. Considering the results obtained when comparing the samples below, it can be assumed that the different perception of an electric scooter is due to the differences in cognitive and emotional components of the attitude, while the behavioral one is approximately at the same level.

### Results for the Sample of Chinese Consumers

When evaluating one's image using the dimensions of the J. Aaker most respondents mentioned their Sincerity (97%), Excitement (83%) and Ruggedness (90.9%). That means that the most respondents perceive themselves as open, friendly, energetic, modern, freedom-loving, and persistent people. Since the number of women in the sample of Chinese respondents significantly exceeds the number of men, it is checked whether this result is related to the gender of the respondents. At the tendency level, it can be noted the tendency of women to give themselves higher marks on the Sincerity dimension (0.16 at *p* < 0.05). Using Student's *t* test, assessments of images of oneself and an electric scooter were compared according to the dimensions of J. Aaker's method, given by respondents of both genders: no significant differences were found between the groups. The groups of men and women also do not differ significantly in terms of adherence to electric scooters.

The image of an electric scooter represented in the minds of Chinese consumers is characterized by Sincerity and Ruggedness (see Table 3). It is according to these dimensions of the method of J. Aaker the majority of respondents (87.9 and 89.7%, respectively) gave high marks to the electric scooter. To the least extent, it is characterized by Sophistication and Competence—high marks for these parameters were given by 48.5 and 64.8%, respectively. This data indicates that the electric scooter is perceived by Chinese consumers as a practical, convenient vehicle that provides the consumer with mobility and comfort. High scores on the Ruggedness dimension indicate that the electric scooter is perceived as sporty and dynamic (Antonova and Morozova, 2015). Low scores on the Sophistication dimension are since the electric scooter is perceived by the Chinese not as a fashionable accessory, but as a clear and convenient technique in everyday life, and low scores on the Competence dimension may be due to its risk of injury, perceived as unreliable.

**Table 3:** Characteristics of electric scooter personality perceived by Chinese consumers.

**E-scooter**	**Sincerity**	**Excitement**	**Competence**	**Sophistication**	**Ruggedness**
% of respondents who gave high marks	87.9	70.3	64.8	48.5	89.7

As can be seen from the initial data analysis (see Table 4), the assessment of respondents' identity and perceived individuality of electric scooters are very close. This suggests that respondents perceive the electric scooter as a product close to themselves, reflecting their identity. Mathematical and statistical analysis confirms this data: statistically significant weak correlations between self-images and the electric scooter at the level of *p* < 0.01 were found on all dimensions of the J. Aaker method.

**Table 4:** Correlations between the corresponding scales of the J. Aaker method “Brand Identity” and modifications for measuring identity.

**Consumer**	**E-scooter**	**Sincerity**	**Excitement**	**Competence**	**Sophistication**	**Ruggedness**
Sincerity		0.24^**^				
Excitement			0.38^**^			
Competence				0.37^**^		
Sophistication					0.43^**^	
Ruggedness						0.35^**^

** *p < 0.01*.

Correlations between self-images and the electric scooter are weak, but it is interesting, the highest correlation was found on the Sophistication dimension—the dimension that has the lowest values when respondents characterize the personality of the electric scooter. That is, respondents who feel refined, charming, endow these qualities and an electric scooter. Correlation analysis showed a weak negative relationship between gender and the difference between oneself and electric scooter images on the Sophistication dimension (0.29 at *p* < 0.01): in men, the image of oneself and the image of an electric scooter on the Sophistication dimension is more consistent than in women.

#### Discussion and Analysis of the Results

Testing the hypothesis about the correlation between the image of oneself and the image of a product as a factor of its preference.

To test the hypothesis about the correlation between the difference in self-image and the electric scooter with the loyalty to this type of transport, the delta was calculated for each of the dimensions of J. Aaker's methodology (Sincerity, Excitement, Competence, Sophistication, and Ruggedness). Then these values were taken modulo, and the Spearman criterion was used to check the correlation with the loyalty and actual use of the electric scooter. Loyalty to an electric scooter, which is understood as a desire to purchase it in the future, is weakly negatively associated with the difference in images on the dimension Sincerity (−0.20^*^), Excitement (−0.16^*^) at *p* < 0.05, and Competence (−0.22^**^) at *p* < 0.01. Interestingly, in addition to the Sincerity and Excitement dimensions, on which most respondents gave themselves high marks, the Competence dimension is also important. This suggests that the perceived similarity between themselves and the electric scooter in terms of Intelligence, Reliability, and Success is related to the loyalty to this type of transport.

Thus, the hypothesis about the correlation between the difference in the images of oneself and the electric scooter with the adherence to this type of transport has been partially confirmed.

### Results for the Sample of Russian Consumers

The image of an electric scooter, represented in the minds of Russian consumers, is characterized by Excitement and Ruggedness. It is on these dimensions of the methodology of J. Aaker that most of respondents (58.7 and 52%, respectively) gave high marks to the electric scooter. Sophistication and Competence are the least characteristics for this sample—high marks for these parameters were given by 26 and 28.6%, respectively. These data indicate that the electric scooter is perceived by Russian consumers as a modern, bright vehicle that gives a feeling of freedom and independence. Low scores on the Sophistication dimension are probably because the electric scooter is not yet a fashionable accessory for Russians.

When assessing their own image on the dimensions of J. Aaker's methodology, most of respondents noted their Sincerity (74%), Competence (76%) and Ruggedness (77.3%). That is, most of respondents recognize themselves as people who are practical, friendly, successful, and freedom-loving.

As can be seen from the initial analysis of the data, there is no contradiction between the identity of the respondents and the perceived identity of the electric scooters, however, they differ. This suggests that the respondents do not perceive the electric scooter as a product close to them, reflecting their identity.

This assumption was also confirmed by statistical analysis: no statistically significant correlations were found between the identity of the respondents and the individuality of electric scooters.

#### Discussion and Analysis of the Results

Testing the hypothesis about the connection between the image of oneself and the image of a product as a factor of its preference.

To test the hypothesis that the similarity between the image of oneself and the perceived individuality of the product as a factor of the consumer's loyalty to this product, the delta between these two dimensions was calculated for each of the variable of the J. Aaker methodology (sincerity, excitement, competence, sophistication, and ruggedness). Then we took these values to build modulo, and used the Spearman test, to check the relationship between this delta and actual use of the electric scooter. Loyalty to an electric scooter, which is understood as a desire to acquire it in the near or distant future, is weakly negatively associated with the difference in images on the ruggedness dimension (−0.28) at *p* < 0.01. Ruggedness is the only dimension that is significant both for the identity of the respondents and for the personality of an electric scooter. Accordingly, the discrepancy on this dimension leads to a lower adherence to this mode of transport.

Thus, the hypothesis about the correlation between the difference in the images of oneself and the electric scooter with the adherence to this type of transport was partially confirmed in both the Chinese and Russian samples.

This allows us to conclude that the approach proposed by N. Antonova to the study of psychological factors that determine consumer loyalty to a brand or product is highly heuristic. The assumption that the consumer's identity is related to the product's personality, as well as that the consistency of these images affects the consumer's loyalty to this product, was confirmed in this study.

Thus, based on the results of this study, the following conclusions can be drawn:

(1) The consumer behavior of Russians and Chinese when choosing transport is different. The Chinese are more likely to use private transport, while the Russians are more likely to use public transport. Chinese consumers are characterized by a higher level of satisfaction with their choice and a pronounced focus on family approval, that is not the same for Russians.
(2) The self-image of Russian and Chinese consumers is different. At the same time, the Chinese perceive themselves as sincere, courageous, and emotionally bright people. Russians' self-image is less differentiated, and they tended to give themselves an average score when evaluating themselves. Along with ruggedness, Russian respondents noted their competence.
(3) The image of an electric scooter represented in the minds of Russian and Chinese consumers is different, and the respondents from China have this image significantly closer to their self-image than the respondents from Russia. Chinese consumers perceive the electric scooter much more emotionally than Russian ones, correlating it with their personality, accepting it as part of their image. However, the differences in the emotional component of the attitude toward an electric scooter are not related to the behavioral component—the level of loyalty to this type of transport does not significantly differ among Chinese and Russian consumers.

## Conclusion

The influence of cultural context on consumer attitudes is undeniable. However, the nature of this influence and its intensity will be different depending on a variety of external factors. This study attempted to cross-culturally analyze the consumer attitudes of Russians and Chinese using the example of their attitude to an electric scooter. The individuality of the product perceived by consumers is considered through the prism of personal identity.

One of the objectives of the study was to identify the correlations between the consumer's identity and the perceived individuality of the product, as well as to analyze cross-cultural differences that affect this correlation. Based on the results obtained, one can say that the correlation between the consumer's identity and a product's personality is one of the factors of the cognitive and emotional components of consumer loyalty to a product. Having carried out a cross-cultural analysis of the data, the self-image of Russian and Chinese respondents differs both in the set of leading personality traits and in the intensity of their expression was found. Differences in self-images are related to differences in the perception of a product.

## Data Availability Statement

The original contributions presented in the study are included in the article/supplementary materials, further inquiries can be directed to the corresponding author.

## Ethics Statement

The studies involving human participants were reviewed and approved by Ethics Committee of Doctoral School of Psychology, HSE University (Moscow, Russia, www.hse.ru). Written informed consent for participation was not required for this study in accordance with the national legislation and the institutional requirements.

## Author Contributions

FZ conducted the collection of data and wrote the paper. The author confirms being the sole contributor of this work and has approved it for publication.

## Conflict of Interest

The author declares that the research was conducted in the absence of any commercial or financial relationships that could be construed as a potential conflict of interest.

## Publisher's Note

All claims expressed in this article are solely those of the authors and do not necessarily represent those of their affiliated organizations, or those of the publisher, the editors and the reviewers. Any product that may be evaluated in this article, or claim that may be made by its manufacturer, is not guaranteed or endorsed by the publisher.
